# Chemopreventive and Anticancer Role of Resveratrol against Oral Squamous Cell Carcinoma

**DOI:** 10.3390/pharmaceutics15010275

**Published:** 2023-01-13

**Authors:** Giuseppe Angellotti, Giulia Di Prima, Elena Belfiore, Giuseppina Campisi, Viviana De Caro

**Affiliations:** 1 Department of Surgical, Oncological and Oral Sciences, University of Palermo, Via L. Giuffrè 5, 90127 Palermo, Italy; 2 Department of Biological, Chemical and Pharmaceutical Sciences and Technologies, University of Palermo, Via Archirafi 32, 90123 Palermo, Italy

**Keywords:** resveratrol, polydatin, oral squamous cell carcinoma, polyphenols, chemoprevention, anticancer, antimetastatic, adjuvant, natural compound

## Abstract

Oral squamous cell carcinoma (OSCC) is one of the most prevailing and aggressive head and neck cancers, featuring high morbidity and mortality. The available conventional treatments suffer from several adverse effects and are often inefficient in terms of their survival rates. Thus, seeking novel therapeutic agents and adjuvants is of the utmost importance for modern society. Natural polyphenolic compounds have recently emerged as promising chemopreventive and anticancer agents. Specifically, the natural compound resveratrol (RSV) has recently gained momentum for this purpose. RSV is useful for treating OSCC due to its antiproliferative, antimetastatic, and proapoptotic effects. Additionally, RSV acts against tumor cells while synergically cooperating with chemotherapeutics, overcoming drug resistance phenomena. Despite these wide-spectrum effects, there are few specific investigations regarding RSV’s effects against OSCC animal models that consider different routes and vehicles for the administration of RSV. Interestingly, an injectable RSV-loaded liposome-based formulation was proven to be effective against both in vitro and in vivo OSCC models, demonstrating that the development of RSV-loaded drug delivery systems for systemic and/or loco-regional applications may be the turning point in oral cancer treatment, leading to benefits from both RSV’s properties as well as from targeted delivery. Given these premises, this review offers a comprehensive overview of the in vitro and in vivo effects of RSV and its main derivative, polydatin (PD), against OSCC-related cell lines and animal models, aiming to guide the scientific community in regard to RSV and PD use in the treatment of oral precancerous and cancerous lesions.

## 1. Introduction

Oral cancers represent the most common head and neck cancers, and about 90% of their cases are histologically defined as squamous cell carcinomas. The onset of this kind of tumor is multifactorial, starting from changes in the normal mucosa and evolving into cancer lesions and then metastasis [1]. Among the risk factors, the abuse of alcohol and tobacco consumption are considered the main leading causes since they are proinflammatory, and it is well-known that the development of oral cancer is closely related to several inflammation pathways [2]. Despite the available therapeutic strategies (e.g., chemotherapy, radiotherapy, and surgery) having been greatly improved over the past few decades, the actual main shortcomings concern the improvement in patients’ survival rates, since they are still below 50% in clinical cases [3]. For this reason, the identification of novel therapeutic agents as well as the development of new therapeutic approaches, aimed at both treatment and chemoprevention, are mandatory. In recent years, the scientific community has extensively focused its attention on the effectiveness of several natural plant-derived compounds that have demonstrated interesting chemopreventive and therapeutic properties against pancreatic and hepatic cancers (e.g., curcuminoids), tumors affecting the gastrointestinal tract (e.g., catechins), breast and prostatic cancers (e.g., indole compounds), melanoma (e.g., apigenin and β-carotene), and oral cancers [4,5,6]. Specifically, several naturally occurring compounds have been shown to possess promising efficacy against oral cancer cells by interfering with the cell cycle, inducing early apoptosis and affecting invasion into other tissues and organs, thus interfering with the metastasis process [7]. Furthermore, these natural actives were generally characterized by low costs, the absence of systemic toxicity, fewer side effects when compared to standard therapies, and a certain capability of enhancing conventional anticancer drugs’ effects. The latter aspect makes these phytocompounds excellent candidates for the treatment of aggressive tumors, such as oral cancers [8]. Among the various investigated natural biomolecules, polyphenols have recently gained considerable interest due to their wide-spectrum properties being potentially useful in the treatment of cancers (e.g., antitumor, antioxidant, antiproliferative, anti-inflammatory, and immunomodulatory) [9]. However, their unfavorable physicochemical properties (e.g., lipophilicity leading to low water solubility) and susceptibility to oxidation and chemical degradation due to pH, light exposure, and high temperature compromise their bioavailability, administrability, and handling, thus limiting their clinical use [10]. Among polyphenols, resveratrol (RSV) has recently emerged as an effective molecule against oral squamous cell carcinoma (OSCC). It should be highlighted that the literature fully reports on the use of RSV as an anticancer agent against various cancer types [11], while its specific employment in the treatment of OSCC is still a little-traveled road. Indeed, although in vitro studies corroborate the potentiality of RSV against OSCC, few in vivo studies report on the treatment of OSCC with RSV using different routes of administration and vehicles. Thus, a comprehensive review that aims to highlight the role of RSV in the treatment of OSCC, as the most prevalent form of oral cancer, is still needed in order to evidence its potential for the scientific community. Based on these considerations, this review aims to give guidance to researchers on the potential use of RSV as a chemopreventive and adjuvant active in the treatment of OSCC by providing a collection of recent literature exploring the in vitro and in vivo effectiveness of RSV against OSCC.

## 2. Methodology

The following criteria were applied to select the papers to be included in the present review: in vitro studies performed only against cancer cell lines related to OSCC; in vivo studies performed only in animal models with OSCC-related xenograft or tumor induction, supported by well-described data and published in high-level journals. All of the selected studies are available in the English language and reported in vivo and in vitro studies regarding the evaluation of RSV and its derivative compounds, such as polydatin (PD). The exclusion criteria were, in contrast, the following: studies only available in a native language different from English; in vitro and in vivo studies published in journals without a peer review process; in vivo studies that did not report the ethical guidelines; in vitro and in vivo studies performed on cell lines which are not directly related to the OSCC, as well as in animal models with no OSCC xenograft; and in vitro as well as in vivo studies aimed at evaluating other antioxidants or polyphenols, or phytochemicals generally or complex matrices (even if these included RSV in their bioactive pool). The literature search was carried out in different databases, such as PubMed and Google Scholar, and was aimed at collecting papers published in the last decade. After the selection of the studies, the following data were collected: in vitro chemopreventive and anticancer effects of RSV and PD in terms of cytotoxic, proapoptotic, and antimetastatic properties; in vivo results in animal models.

## 3. Oral Squamous Cell Carcinoma (OSCC)

OSCC is a neoplasm that originates from oral keratinocytes mutating in malignant cells, and it is one of the most common oral cancers in terms of morbidity and mortality worldwide [12,13]. Various sites of the oral cavity can be affected by this tumor, such as the lips, the tongue, and the floor of the mouth [14]. The most common risk factors leading to OSCC development seem to be related to the abuse of alcohol and cigarette consumption. In particular, the chemical substances produced when smoking tobacco (benzopyrenes and nitrosamines) diminish the immune responsiveness of the oral environment as well as compromise the DNA of cells, promoting carcinogenesis [15]. Consequently, at the beginning of the carcinogenesis process, some lesions appear in the epithelium. Lesions could be histologically classified in terms of observed changes in the affected tissue, such as keratinocyte aspect modifications or hyperplasia. These alterations characterize the stage of cancer before the metastasis process [16]. Unfortunately, OSCC is mainly diagnosed at a very late stage [17] thus badly compromising the probability of survival and reducing patients’ quality of life. Consequently, the success of OSCC treatment depends on an appropriate and quick intervention at the first stage of the tumor [18]. The conventional therapeutic approaches are based on surgery, radiotherapy, systemic chemotherapy, or combinations thereof [19]; however, these strategies are often aggressive, thus negatively affecting patients’ quality of life, while likewise being often unsatisfactory in terms of survival rate. Considering the high incidence of OSCC, it is still necessary to identify novel bioactive compounds as well as to design new drug delivery systems aimed at increasing the efficacy of conventional treatments, minimizing their adverse effects and thus allowing their drawbacks to be overcome. A winning strategy with which to reduce the chemo- and radiotherapy-related side effects, whilst also improving and empowering their efficacy, could consist of the administration of natural molecules as chemopreventive and adjuvant agents. Cancer chemoprevention is based on the use of nutraceuticals or phytochemicals to reverse carcinogenesis before the metastasis phase occurs. This can be achieved thanks to their ability to block key events of tumor initiation and/or inhibit the ability of cancer cells to migrate to other tissues, thus reversing the premalignant stage.

Chemoprevention is a promising strategy, since it has been demonstrated that adequate treatment during the early stage of cancer could positively affect the carcinogenesis pathways [20,21]. Numerous chemicals are used as chemopreventive agents, such as antiestrogens, antiandrogens, anti-inflammatories, and vitamins. Among them, phytochemicals have been demonstrated to be efficacious and characterized by low side effects and costs [20,22]. In particular, polyphenols have recently been under the spotlight due to their wide-spectrum therapeutic properties, among which the chemopreventive and anticancer activities should be pointed out. Indeed, recent findings evidenced polyphenols as chemopreventive agents and effective adjuvants with which to treat oral cancerous or precancerous lesions, in order to suppress or reverse tumor progression [23].

## 4. Resveratrol (RSV) and Polydatin (PD)

**Figure 1:**
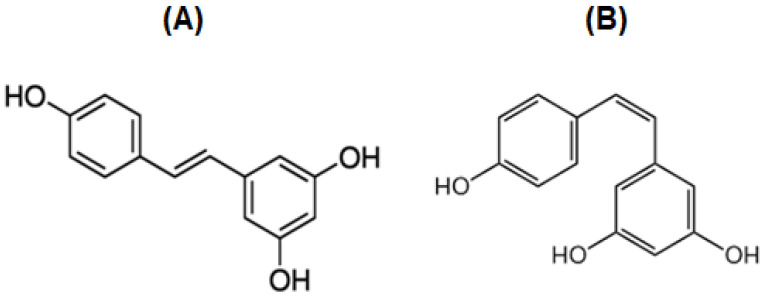
Chemical structure of *trans* (**A**) and *cis* (**B**) RSV.

RSV is a polyphenol-based compound belonging to the phytoalexin group. It is widely found in red grapes, berries, and peanuts, and it is naturally synthesized by plants in response to external stimuli, such as microbiological infections [24]. Chemically, it exists in two isomeric forms (*cis* and *trans*, Figure 1), but only *trans*-RSV is biologically active. The isomerization to the *cis* form is the result of various instability phenomena (e.g., UV irradiation; exposure to alkaline pH) which might occur, for example, during grape juice fermentation.

**Figure 2:**
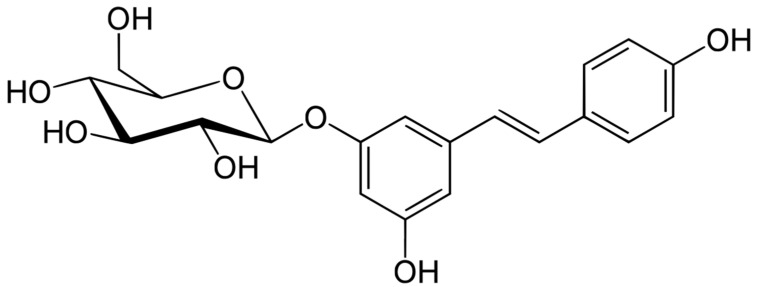
Chemical structure of PD.

Furthermore, RSV also exists as dimers, trimers, and glucosides. In this case, the most representative derivative is PD (Figure 2).

The presence of β-d-glucosyl residue at position 3 confers favorable physiochemical characteristics (e.g., enhanced water solubility) while preserving and maintaining RSV’s beneficial properties [25]. Among the various polyphenols in nature, RSV has recently been the most studied due to its antioxidant [26], anti-inflammatory [27], antiaging [28], cardioprotective [29], and bone-regenerative [30] properties. For these reasons, it is widely used as an active ingredient or adjuvant in cosmetic and pharmaceutical products [31]. In recent years, the scientific community has focused on the antitumor, antiproliferative, and chemopreventive effects of RSV that allow its application in the treatment of various types of cancers. Although the real process of RSV-mediated chemoprevention has not been fully and clearly understood yet, several mechanisms have been proposed to describe its antitumor activity. Briefly, it acts by inducing cancer cell apoptosis through the activation of multiple pathways, and in particular by modulating the activity of the mitogen-activated protein kinase (MAPK) and p53 protein pathways [32]. Furthermore, RSV has been shown to reduce the aptitude of cancer to metastasis in two ways: i) the inhibition of the gene expression of extracellular matrix metalloproteinases (e.g., MMP-2 and MMP-9) involved in tumor invasiveness; ii) the suppression of vascular endothelial grow factor (VEGF) expression, leading to the reduced formation of new tumor-specific blood vessels [33]. Therefore, it is evident that RSV could be a key molecule in the treatment of neck and head cancers, such as OSCC.

## 5. In Vitro and In Vivo Chemopreventive and Anticancer Activities of RSV and PD against OSCC

### 5.1. In Vitro Studies

The role of RSV as a chemopreventive and anticancer agent has been extensively investigated in vitro against several OSCC cell lines, and various mechanisms of action were observed, proposed, and verified.

Atienzar et al. evaluated the effect of RSV against OSCC by treating the PE/CA-PJ15 human oral squamous carcinoma cell line with various RSV concentrations (5, 10, 25, 50, and 100 µM) for 24, 48, and 72 h. They found that RSV displayed concentration-dependent cytotoxicity. In detail, the highest reduction in terms of cell viability, together with the best increase in cell apoptosis, were achieved by using RSV concentrations of 50 and 100 µM. Furthermore, they investigated the mechanism by which RSV affects the cell cycle. At the mentioned higher concentrations, RSV emerged as being capable of changing the regulation of the G0-G1, G2-M, and S phases, thus modifying the duration of each phase. In particular, it is interesting to note that, after 24 h of treatment, RSV induced the prolongation of the S phase rather than the other ones. Finally, a scratch test was used to study RSV’s impact on cell migration. At concentrations between 25 and 100 µM, RSV was found to reduce cell migration, thus suggesting its potential role in hindering the metastasis process [34].

Yu et al. employed the SCC-VII, SCC-25, and YD-38 cell lines, treating them with increasing RSV concentrations (0.1–1.5 μg/mL). In particular, the inhibitory effect of RSV against the cell proliferation rate was confirmed to be concentration-dependent. Moreover, the obtained IC50 values (48 h treatments) against SCC-VII, SCC-25, and YD38 OSCC were 0.5, 0.7, and 1.0 μg/mL, respectively. In addition, a 48-h treatment with RSV caused cells to interrupt their cell cycle. This effect was probably due to RSV’s ability to enhance the expression of the Myt1 protein, which induced the phosphorylation of the cdc2 protein, in turn controlling G2/M phase progression. Additionally, RSV was significantly found to induce apoptosis in all three OSCC cell lines considered [35].

In 2001, Shan’s group analyzed the effect of RSV on the adhesion, migration, and invasion of cancer cells by using KB cells as a model with which to examine the metastatic mechanisms in OSCC. A concentration-dependent reduction in cell adhesion was observed after treatments with 25, 50, and 100 µM of RSV over a period of 5 h. The maximum concentration analyzed decreased cell adhesion by 49.92% and 58.21% after 1 and 2 h of treatment, respectively, when compared to the control. Furthermore, the adhesion and migration processes were significantly reduced by increasing RSV concentration, but only the highest concentration evaluated (100 µM) had a significant inhibitory effect [36].

According to a latter work, Kim and colleagues studied the mechanism of KB cell death after treatment with RSV. They highlighted that RSV affects cell viability in both a time- and concentration-dependent manner. Cells were exposed to increasing concentrations of RSV (from 30 to 300 µM) up to 72 h. The obtained IC50 values were 197.4 and 63.3 µM after 12 and 72 h, respectively. To determine whether apoptosis was the main cause of KB cell death, analyses regarding DNA fragmentation and caspase activation were conducted on cells treated with RSV concentrations of 30 and 100 µM for 48 h. The authors found that RSV caused internucleosomal DNA fragmentation. Additionally, using immunoblotting tests, they proved its role in promoting both the cleavage of procaspase-3 and caspase-3 as well as the proteolytic cleavage of procaspase-7 and procaspase-9. These results suggested that one of the possible anticancer mechanisms of RSV against OSCC is related to apoptosis induction via caspase interaction [37].

Kim et al. performed, in 2018, a deep and interesting study regarding the chemopreventive effect of RSV against three different OSCC cell lines: Cal-27, SCC-25, and SCC15. These were treated with increasing concentrations of RSV (10–500 µM) until different time points: 24, 48, and 72 h. Against the Cal-27 cell line, cell viability was reduced by 33.9% after a 24 h treatment with the highest RSV concentration tested, while only minimal reductions were observed at the lower concentrations. In reality, the obtained IC50 values were 100 µM for a 24 h treatment against Cal-27, 200 µM for a 72 h treatment against SCC-25, and 300 µM for a 72 h treatment against SCC-15. In addition, they investigated the proapoptotic properties of RSV, highlighting that the number of apoptotic cells was higher than that of the control group, and, in particular, the Cal-27 cell line displayed the maximum value. Furthermore, they found that the mechanism involved in the onset of RSV-induced apoptosis was linked to multiple pathways: the downregulation of MMPs, the activation of the Bax and Bak proteins, the reduction in bcl-2 and bcl-XL expression, the induction of cytochrome c release from mitochondria, and the activation of the caspase proteins (caspase-3 and caspase-9). These results suggested that RSV acts as an antitumoral agent in OSCC cell lines by inducing apoptosis through the activation of mitochondrial pathways and caspases modulation. Finally, they further described a key property of RSV: it was demonstrated to be able to decrease cell migration and invasion via the inhibition of the epithelial–mesenchymal transition (EMT) transcription factor, specifically against Cal-27 cells [38].

Kim et al. focused on the ability of RSV to counteract the invasion of cancer cells, investigating this effect against YD-10B oral squamous carcinoma cells stimulated by lysophosphatidic acid (LPA) as a well-known proinvasion agent. The experiments were conducted by pretreating a group of cells with LPA only (control) and other groups with both LPA and RSV 25 µM for 1 h. The number of invading cells was quantified after a further 12 h of incubation. As a result, LPA significantly stimulated cancer cells’ invasion aptitude, as emerged from the observed overexpression of the two most relevant EMT transcription factors (TWIST and SLUG). In contrast, the RSV-based treatment caused the downregulation of all of these factors, thus decreasing the number of invading cells compared to those counted in the control group [39].

Further investigations regarding the antimetastatic properties of RSV against HNSCC cells, one of the most aggressive OSCC types, were conducted in 2020 by the Kim research group. This type of cell line is characterized by the overexpression of the Rab coupling protein (RCP), which promotes cancer cells’ invasion via EMT factors, the Zeb-1 protein, and MT1-MMP expression. Three cell lines (YD-9, YD-10B, and YD-38) were treated with increasing concentrations of RSV up to 25 µM. RSV was highlighted to negatively affect the invasiveness of all of the treated cell lines in a dose-dependent manner. Moreover, the mechanism involved was also determined: RSV was proven to downregulate the MT1-MMP protein, block the recycling process of β1 integrin to the plasma membrane, and thereby inactivate the epidermal growth factor receptor (EGFR), also reducing the signaling cascade leading to Zeb1 expression. These results confirm the antitumor properties of RSV related to its cytotoxicity. They also show that RSV is a powerful antimetastatic compound thanks to its mechanisms of modulation of both cell adhesion and invasion [40].

Shang et al. examined the ability of RSV to suppress the MAGEA12/Akt signaling pathway against OSCC by using Cal-27 cells as a model. This pathway is involved in a complex series of processes leading to tumor proliferation, migration, and invasion. The researchers observed that MAGEA12 overexpression significantly increased the viability of cells when compared to the control group (identical cells but without MAGEA12 overexpression) over time. Furthermore, after subjecting cells to RSV-based treatments (10, 20, 50, and 100 μM) for 48 h a significant dose-dependent reduction in the MAGEA12/Akt cascade was observed, and the RSV IC50 was equal to 50 μM. However, the inhibitory effect of RSV slightly decreased against cells overexpressing the MAGEA12/ark proteins. The results suggested that MAGEA12/ark pathway downregulation might contribute to the anticancer effect of RSV; however, additional studies were required [41].

The team headed by Masuelli studied the in vitro and in vivo (see below) effects of RSV on head and neck squamous cell carcinomas when coadministered with curcumin (CUR) in order to evaluate any occurring synergic effects between the two polyphenols. Three different OSCC cell lines (Cal-27, SCC-15, and FaDU) were treated with RSV, CUR, or a combination thereof, at various concentrations (6.2, 12.5, 25, and 50 μM) for 48 h. They highlighted that the combination of these polyphenols allows a significant dose-dependent reduction in terms of cell viability when compared with the results obtained by RSV and CUR singularly administered at all of the tested concentrations [42].

The resistance of OSCC to chemotherapy, particularly when administering cisplatin, is currently a big challenge. For this reason, Chang et al. explored the role of RSV against a Cal-27 cisplatin-resistant cell line in terms of proliferation and metastasis, while also aiming at pointing out the mechanism of action involved. Cells were treated with RSV 10, 25, 50, and 75 μM for 24 h; however, among all of the tested concentrations only the highest produced a minimum cytotoxic effect, while lower doses were not able to affect the viability of the studied cancer cells. However, even the non-toxic RSV concentrations significantly reduced cells’ invasion and migration abilities in a dose-dependent manner when compared to controls. RSV 50 μM treatment definitely stopped cell migration at both 12 and 24 h, as observed by the scratch test. Furthermore, due to RSV treatment, the strong inhibition of extracellular signal-regulated kinases (ERKs) and p-38 phosphorylation was observed without a reduction in their expression, while the expression of MMP-2 and MMP-9 was significantly downregulated. These pieces of evidence suggest the important role of RSV as an adjuvant and chemopreventive agent against cisplatin-resistant OSCC forms, diminishing the metastasis process despite the low cytotoxicity [43].

Among the antineoplastic agents administered to treat OSCC, monoclonal antibodies such as cetuximab are the most promising tools nowadays. However, they cannot be used for long-term treatments due to cancer cell resistance phenomena which quickly occur because of certain genetic mutations (e.g., regarding EGFR and KRAS). For this reason, Uzawa et al. investigated the in vitro and in vivo effects (see below) of RSV in cetuximab-resistant OSCC cell lines and xenografted mice models, respectively. Three different OSCC cell lines (SAS, Sa3, and HSC-3) were treated with cetuximab to induce mutations and, thereby, resistance. After treatment, the resulting cetuximab-resistant cells (denominated SAS-R, Sa3-R, and HSC-3-R) were characterized by no significant differences in terms of EGFR expression while phosphorylated ERK1/2 levels were increased. Based on these results, researchers further investigated the mechanism involved in the onset of cetuximab resistance via a gene expression microarray. The obtained data showed that the urokinase-type plasminogen activator receptor (uPAR) was upregulated in all of the considered cell lines. This gene is strongly associated with the regulation of the EGFR/p-ERK1/2-related signal pathway. Afterwards, the so-transformed cells were treated with RSV 20 μM for 24 h, resulting in the downregulation of both integrin β1 and uPAR expression, together with reduced cell viability. These results suggested RSV as a valuable agent with which to counteract the overexpression of uPAR and overcome the resistance to cetuximab, thus improving the long-term effectiveness of OSCC therapy [44].

Finally, the antitumoral properties of PD, as the most abundant RSV glycoside derivative, were also explored by Bang et al. in 2021. The Ca9-22 and Cal-27 cell lines were used as OSCC model cells, while keratinocytes were chosen as control normal cells. They were all treated with increasing concentrations of PD up to 2 mM for 24 and 72 h. PD resulted in being compatible with normal control cells, while it killed cancer cells, with IC50 values of 1.15 and 0.95 mM for Cal-27 and Ca9-22 cells, respectively. The cytotoxic effect of PD was related to its ability to stimulate apoptotic and autophagic processes selectively against cancer cells. In particular, the proapoptotic activity was due to several dose-dependent effects against both of the cancer cell lines considered: the condensation and cleavage of nuclei, the release of cytochrome c from mitochondria, the decrease in bcl-2 synthesis, and the increase in Bax expression. Additionally, the treatment with PD 0.25 mM increased the expression of ATG5 and LC3 proteins, thereby inducing autophagy in both Ca9-22 and Cal-27 cells. Finally, PD displayed antimetastatic properties due to the suppression of Snail and Slug proteins at the cell junction level, leading to the enhanced expression of E-cadherin and the downregulation of N-cadherin [45]. These results confirmed that the presence of the glycoside moiety did not alter the anticancer properties of RSV, thus confirming PD as a valid alternative to RSV in OSCC treatment and chemoprevention.

### 5.2. Summary of the Employed Cell Lines, Considered Markers, and General Overview of the in Vitro Results

A summary of some crucial key points might be useful, especially given the complexity of the literature material in terms of OSCC-related cancer cell lines, pathways to consider, and markers to follow.

The OSCC-related cancer cell lines used in the reported studies were as follows: PE/CA-PJ15; SCC-VII; SCC-25; SCC-15; YD-10B; YD-9; YD-38; KB; SAS; Sa3; HSC-3; FaDU; Ca9-22; Cal-27; Cal-27-overexpressing MAGEA12; and cisplatin-resistant Cal-27. As a valid alternative, the literature also reports on the employment of 3D cultures based on biopsy samples, aiming to overcome the limitations of simpler 2D models and each cell line [46,47].

A general dose- and time-dependent cytotoxic effect also widely emerged (IC50 values ranging from 50 to 300 μM) via the downregulation of the MAGEA12 pathway, which, when activated, led to promoted cancer cell viability [41]. Additionally, the following effects as well as related pathways and markers were considered:

- The modulation of the cell cycle via the prolongation of the S phase and the interruption of the cell cycle due to the enhanced expression of Myt1 [34,35].
- Proapoptotic effect due to internucleosomal DNA fragmentation; the cleavage of procaspase-3, -7, and -9, as well as caspase-3; the downregulation of MMPs; the activation of Bax and Bak proteins; the reduction in bcl-2 and bcl-XL expression; the activation of caspase-3 and -9; and the release of cytochrome c from mitochondria [37,38,45].
- Antimetastatic effects due to reduced cell adhesion (even after short, 1–5 h, treatments) and migration. Particularly, after LPA (proinvasion agent) pretreatment, the antimetastatic effect emerged as being related to the promotion of EMT transcription factors (e.g., TWIST and SLUG) [36,38,39,40,43,44].
- Adjuvant activity in combination with conventional chemotherapeutic molecules (e.g., cisplatin and cetuximab) and the ability to overcome the occurring drug resistance phenomena via ERK inhibition, p38 phosphorylation, and the reduction in MMP-2 as well as -9 [42,43,44].

The data collected and reported in this review certainly highlight RSV’s anticancer and chemopreventive effects against a wide variety of cancer cell lines associated with OSCC (Table 1). As reported, its effectiveness is multifactorial due to interactions with several biological pathways that regulate cellular differentiation and apoptosis, as well as the metastatic process. It is interesting to note that the main antimetastatic mechanism of RSV is related to its ability to inhibit or block the invasion of cancer cells by promoting EMT transcription factors and downregulating MMPs. These properties make it perfectly capable of fighting the main characteristics of oral cancers and particularly OSCC, which is often characterized by an unfavorable prognosis largely due to its high metastatic power. To highly benefit from RSV’s effects, it should be crucial to treat early stage OSCC patients by providing RSV as an adjuvant to conventional anticancer treatments, thus blocking the metastatic process and improving patients’ quality of life as well as maybe increasing the survival rate.

### 5.3. Safety of RSV Administration

The above paragraphs reported all of the collected in vitro studies regarding the role of RSV against OSCC-related cancer cell lines; control studies against healthy cells were not performed in the mentioned articles. The study by Bang et al. [45] constitutes the only exception, and reports cytotoxicity/cytocompatibility assays against both cancer cells (Ca9-22; Cal27) and keratinocytes (selected as healthy control cells) when administering polydatin. These studies highlighted the selective cytotoxic effect against Ca9-22 and Cal27 cells while displaying cytocompatibility with healthy cells.

However, there are several articles confirming RSV cytocompatibility with various healthy cell types. As with many other natural compounds, RSV exerts a biphasic effect. Indeed, several studies have demonstrated that high doses of RSV lead to a decline in cell viability, while low doses could determine a proliferative effect [48,49]. Rocha et al. investigated the effect of RSV from 25 to 500 µM, for 24 h, against immortalized fibroblasts (HaCaT cell lines). Via an MTT assay, they observed a decline in cytocompatibility from a 100 µM RSV solution (cell viability of about 70%) and an IC50 value of 174.5 µM [50].

The protective role against the oxidative stress related to doxorubicin administration was assessed by Ivanova et al. against both leukemic and normal lymphocytes obtained from healthy human donors. Furthermore, normal lymphocytes were incubated with RSV solutions up to 50 µM for 48 h, always being cytocompatible [51].

Additionally, in 2014 Orihuela-Campos et al. highlighted the protective role of RSV in human gingival fibroblast (HGF-1) against oxidative stress. In brief, HGF-1 cells were pretreated with H_2_O_2_ to induce oxidative stress and suddenly treated with RSV solutions (25, 50, and 75 µM) for 48 h. The protective role of RSV as well as its proliferative effect emerged. Additionally, the authors demonstrated the overexpression of collagen gene I, thus suggesting a promising application for wound healing purposes [52]. These results also confirm the potential application of RSV application into the oral cavity without any risk.

In conclusion, it is worth underlining that RSV can be considered safe against healthy cells.

### 5.4. In Vivo Studies

As collected, the literature reports in vitro studies; nowadays, only few in vivo studies are available concerning the specific topic discussed here

In particular, the chemopreventive effect of RSV on OSCC onset was evaluated in vivo by Berta et al. following the local administration of RSV by employing hydroxypropyl-β-cyclodextrin (HPCD) as a vehicle. Syrian golden hamsters were pretreated with dimethylbenzanthracene (DMBA), used as a well-known proneoplastic agent, and then divided into four distinct groups subjected to treatment with just DMBA (control group; group I) or treated two days a week locally with RSV 74.5 mM (dissolved in ethanol; group II) and an RSV–HPCD complex administered in form of cream (group III) or mouthwash (group IV) at the same RSV dose employed in the evaluation of the ethanol solution. As a result, animals treated with RSV showed a relevant reduction in terms of the prevalence and multiplicity of oral preneoplastic lesions (OPLs). RSV was able to prevent and delay about 60–70% of OPL onset when compared to the positive control group. Moreover, the administration of the RSV–HPCD complex strongly promoted a chemopreventive effect. Among the two proposed pharmaceutical forms, the mouthwash highlighted the best efficacy. The main considered parameters were related to the presence and dimension of the exophytic lesions (ExLs). ExLs indicate the progression and type of cancer lesion: small ExLs are generally related to papilloma, while large lesions are observed in OSCC. Accordingly, the control group displayed bigger lesions due to OSCC development, while the groups treated with RSV depicted small ExLs with a decreasing dimension trend going through RSV solution, RSV–HPCD cream, and RSV–HPCD mouthwash. The described results clearly suggest that the topical application of the RSV–HPCD complex could constitute a promising chemopreventive approach to managing OSCC by reducing the progression of preneoplastic lesions [53].

Masuelli et al., already reported in the in vitro study section, also tested the in vivo chemopreventive efficacy of RSV and CUR coadministration. BALB/c mice expressing salivary gland cancer cells of a SALTO type were divided into four groups receiving per os DMSO (control group; group I), RSV (dose: 2 mg; group II), CUR (dose: 2 mg; group III), and a combination of RSV+CUR (dose: 2 mg of RSV + 2 mg of CUR; group IV). The administration was performed two weeks before the insertion of SALTO cancer cells or simultaneously with this event to evaluate any differences. After eight weeks of treatment, the coadministration of RSV+CUR determined the greatest reduction in tumor volume, even though reduction effects were also observed for both RSV and CUR alone. Additionally, almost 33% of the cases treated by the combination of the two polyphenols were characterized by complete cancer regression. Furthermore, the median survival time of mice increased when polyphenols were administered together. However, there were no statistical differences when evaluating their effect as a function of their administration (prior to or simultaneously with SALTO cells). Moreover, it was also confirmed that the coadministration of RSV and CUR is completely safe, since the hematological and clinical parameters were not altered [42].

Uzawa et al. tested the in vitro (see Section 5.1) as well as in vivo effectiveness of RSV against cetuximab-resistant OSCC. Nude BALB / cAnNCrj-nu/nu mice, xenografted with SAS and Sa3 cancer cells resistant to cetuximab (named SAS-R and Sa3-R, respectively), were used as models. The animals were divided into four groups and subjected to treatment with DMSO (control group; group I), cetuximab (group II), RSV (group III), and cetuximab combined with RSV (group IV). Both actives were administered intraperitoneally as follows: cetuximab 10 mg/kg three times weekly and RSV 100 mg/kg daily. The in vivo results confirmed the already-reported in vitro findings. In particular, cetuximab treatment did not show relevant tumor growth inhibition. In contrast, the group treated with RSV highlighted a significant suppression of tumor growth, together with the downregulation of uPAR expression. Additionally, the combination of RSV and cetuximab reduced cancer proliferation without inducing systemic toxicity, displaying a synergic effect in terms of the downregulation of the uPAR/integrinβ1/p-ERK1/2 pathway. These results confirmed that RSV could be considered as a useful adjuvant molecule with which to overcome cetuximab resistance, thus promoting the long-term effectiveness of cetuximab-based OSCC therapy [44]. In vivo data are summarized in Table 2.

## 6. Innovative RSV-Loaded Formulations to Treat OSCC

Despite several papers confirming RSV and PD as anticancer and chemopreventive agents against OSCC, only one article has been published in recent years reporting the development and in vitro/in vivo evaluation of RSV-loaded drug delivery systems.

Zheng et al. recently (2019) proposed RSV-loaded liposomes for the treatment of head and neck squamous cell carcinomas. Liposomes were also formulated by adding a specific targeting dodecapeptide (named GE11) which is able to bind to EGFR, a receptor commonly overexpressed in head and neck squamous cancer cells. Liposome characterization revealed the homogeneity and nanometric sizes of vesicles, high RSV encapsulation efficiency, and sustained drug release behavior. The effectiveness of the proposed formulations (with or without the GE11 peptide) was assessed in vitro by a cytotoxicity assay against the SCC HN cancer cell line (SCC-VII). Cells were treated with free RSV or RSV-loaded liposomes (at an RSV concentration of 50 μg/mL) for 24 h and compared to untreated control cells. As a result, the RSV-loaded liposomes were shown to be more cytotoxic than free RSV, and the GE11-linked liposomes in particular displayed a stronger effect. Based on these promising in vitro results, the authors further performed in vivo studies on SCC-xenografted nude mice that were divided into four groups: subjected to no treatment (control group; group I), systemic treatment with RSV (10 mg/kg three times every three days; group II), or systemic treatment with RSV-loaded liposomes with or without the GE11 peptide (doses corresponding to 10 mg/kg three times every three days; group III and group IV, respectively). While the control group was characterized by uncontrolled tumor growth, the group treated with free RSV depicted a limited reduction in tumor growth. In contrast, the treatment with RSV-loaded liposomes produced significant antitumor effects and, in particular, liposomes containing the GE11 peptide were the most effective ones, causing a two-fold decrease in terms of tumor volume compared with the free RSV group and a three-fold decrease compared to the control group. This effect could be ascribable to the strong affinity of the GE11 peptide toward the EGFR receptor overexpressed in head and neck squamous cell tumors [54]. These results are greatly relevant and confirm the usefulness of innovative drug delivery systems loaded with natural compounds, such as RSV, in establishing advanced therapeutic approaches with which to efficiently treat OSCC.

## 7. Conclusions and Future Perspectives

OSCC is characterized by bad prognoses due to its aptitude to rapidly generate metastasis, thus dramatically invading other districts and organs. This being the case, the administration of efficient treatments is crucial for improving patients’ survival rates. Today, the available therapeutic options include radiotherapy, chemotherapy, and surgery, which suffer from several adverse effects together with an extremely low 5-year survival rate. A powerful strategy for both prevention as well as supporting conventional drug therapies of OSCC could be the administration of naturally occurring compounds, such as polyphenols, and particularly RSV. As reported, RSV has emerged as a potentially useful molecule as it possesses selective dose-dependent cytotoxicity against cancer cells in addition to proapoptotic, antiproliferative, and antimetastatic effects against several OSCC-related cancer cell lines. It has also been proven to possess chemopreventive, anticancer, and antimetastatic properties against OSCC-related animal models. Importantly, RSV has been proven to act synergistically with conventional chemotherapeutic molecules (e.g., cisplatin and cetuximab), also overcoming the eventually occurring drug resistance phenomena. Additionally, PD (the most representative RSV derivative) still maintains RSV pharmacological activities, although higher doses have to be administered to achieve similar effects. In light of the information collected here, RSV could represent an effective opportunity for cancer chemoprevention and support conventional drug treatments.

Moreover, nowadays only one published paper has proposed the administration of RSV-loaded drug delivery systems to systemically treat OSCC; however, a local treatment of the tumor could represent an attractive alternative to systemic ones. Indeed, topical drug administration is generally characterized by several advantages, such as lower required drug doses, the ease of administration, the bypass of the stomach and hepatic metabolism, and high patient compliance as well as adherence to treatment. Concerning this point, several innovative formulations (e.g., lipid nano- or microparticles) have already been proposed for local RSV delivery, especially to treat skin cancers such as melanoma. The feasibility of efficiently administering RSV directly on the skin surface, as well as the usefulness of RSV as an antimelanogenic agent capable of reducing tumor progression, thus inhibiting the metastasis process, have already been widely demonstrated by in vivo studies [55,56,57]. As an example, Ravikumar et al. described the design, preparation, and characterization of RSV-loaded lipid nanoparticles able to reduce tumor size, fight the inflammatory environment, and decrease metastasis aptitude after topical skin administration against mice affected by melanoma [56]. Additionally, various studies have already confirmed the feasibility of applying RSV directly onto the oral cavity. Moreover, some formulations containing RSV and designed to be specifically applied to the oral cavity mucosae have already been proposed for the treatment of periodontitis [58] and inflammatory lesions [59], as well as to improve RSV bioavailability [60]. For cancer treatment and chemoprevention, local drug delivery has been widely proven to be highly effective as it provides therapeutic drug levels directly at the site of action, thereby improving efficacy while minimizing adverse effects. The development of drug delivery systems specifically designed to be administered on the oral mucosa can provide a more targeted and efficient drug delivery option while also being self-administrable and well-accepted by patients. As a consequence, several buccal delivery systems loaded with natural anticancer compounds (e.g., anthraquinones) and drugs (e.g., 5-fluorouracil) have already been proposed [61,62,63,64,65].

However, to date, the development of RSV-loaded drug delivery systems for the local treatment/chemoprevention of oral precancerous or cancerous lesions has not been reported yet. Thus, based on the greatly demonstrated anticancer, antimetastatic, and chemopreventive properties of RSV against OSCC, researchers should investigate the use of this powerful polyphenol to treat the mentioned disease by utilizing a local approach. Indeed, the development of topically administrable RSV-loaded drug delivery systems could be the turning point in oral cancer treatment.

## Figures and Tables

**Table 1:** Summary of the in vitro chemopreventive, anticancer, and antimetastatic effects of RSV and PD against OSCC-related cancer cell lines: tested concentrations, times, employed cells, markers, and general results.

In Vitro Concentrationsand Times	Cell Lines	Markers	Results	Reference
**5, 10, 25, 50, and 100 µM, for 24, 48, and 72 h**	PE/CA-PJ15		Dose-dependent cytotoxicity. Prolongation of the S phase of the cell cycle (50–100 µM). Reduction in cell migration ability (25–100 µM).	Atienzar et al. [34]
**From 0.1 to 1.5 μg/mL,** **for 48 h**	SCC-VII SCC-25 YD-38	Myt1,cdc2 proteins	Dose-dependent cytotoxicity. IC50 values: 0.5–1 μg/mL. Cell cycle interruption (48 h treatment) by the upregulation of Myt1 and the phosphorylation of cdc2. Promotion of cell apoptosis.	Yu et al. [35]
**25, 50, and 100 μM,** **for 5 h**	KB		An RSV concentration of 100 μM significantly reduced cell adhesion and migration.	Shan et al. [36]
**30–300 µM,** **for a maximum of 72 h**	KB	Procaspase-3, -7, and -9, caspase-3	IC50 values: 197.4 and 63.3 µM after 12 and 72 h treatments, respectively. Pro-apoptotic activity by internucleosomal DNA fragmentation as well as the cleavage of procaspase-3, -7, and -9, and caspase-3.	Kim et al. [37]
**10–500 µM,** **for 24, 48, and 72 h**	Cal27 SCC25 SCC15	MPP, Bax, Bak, bcl-2, bcl-XL, cytochrome c, caspase-3, caspase-9, and EMT transcription factor	IC50 values: 100 µM against Cal27 (24 h treatment), 200 µM against SCC15 (72 h treatment), and 300 µM against SCC25 (72 h treatment). Proapoptotic effect by the modulation of several factors: MMP, Bax, Bak, bcl-2 and bcl-XL, cytochrome c, and caspases. Reduction in cell migration by the inhibition of the EMT transcription factor.	Kim et al. [38]
**25 µM,** **for 1 h**	YD-10B	TWIST, SLUG	Downregulation of EMT transcription factors, resulting in the reduction in the number of invading cells when compared with the positive control (LPA).	Kim et al. [39]
**Up to 25 µM**	YD-9YD-10B YD-38	MT1-MMP, Zeb1	Dose-dependent reduction in cell invasion. Suppression of MT1-MMP and Zeb1 expression.	Kim et al. [40]
**10, 20, 50, and 100 µM,** **for 48 h**	Cal-27	MAGEA12/Akt pathway	Dose-dependent reduction in cell viability and the MAGEA12/Akt cascade. IC50 value: 50 µM. Slightly decreased effect against cells overexpressing MAGEA12.	Shang et al. [41]
**Coadministration with CUR at 6.2, 12.5, 25, and 50 μM, for 48 h**	Cal-27 SCC-15 FaDU		Dose-dependent cytotoxicity, which was enhanced with the coadministration of the two polyphenols.	Masuelli et al. [42]
**10, 20, 50, and 75 µM,** **for 24 h**	Cisplatin-resistant Cal-27	ERK, p-38, MMP-2, and MMP-9	Reduced cytotoxicity in cisplatin-resistant cells. Dose-dependent antimetastatic effects. Inhibition of ERK and p-38 phosphorylation, as well as the downregulation of MMP-2 and -9 expression.	Chang et al. [43]
**20 μM,** **or 24 h**	SAS Sa3 HSC-3	EGFR ERK1/2 uPAR	Increased level of phosphorylated ERK1/2. Downregulation of integrin β1 and uPAR expression.	Uzawa et al. [44]
**PD up to 2 mM,** **for 24 and 72 h**	Ca9-22 Cal-27 Keratinocites	Cytochrome c, bcl-2, bax, ATG5, LC3, E-cadherin, N-cadherin, SLUG, and Snail	Dose-dependent cytotoxicity against cancer cells. Cytocompatibility against healthy cells (keratinocites) at the tested concentrations.IC50 values: 1.15 and 0.95 mM for Cal-27 and Ca9-22 cells, respectively. Proapoptotic effect via the release of cytochrome c, decrease in bcl-2 synthesis, and increase in bax expression. Autophagy induction by the stimulation of ATG5 and LC3 expression. Antimetastatic effect by increasing E-cadherin expression and the suppression of Snail and Slug proteins.	Bang et al. [45]

**Table 2:** Summary of the in vivo chemopreventive, anticancer, and antimetastatic effects of RSV against OSCC-related animal models: tested concentrations, times, and general results.

In Vivo Dose and Timing	Animal Models	Results	Reference
**2 mg of RSV, 2 mg of CUR, or 4 mg of an RSV+CUR combination per os two weeks before or simultaneously with SALTO treatment**	BALB/c treated with SALTO cancer cell line	The 33% of the cases treated by the combination of RSV+CUR displayed complete cancer regression, synergic effects, and safety.	Masuelli et al. [42]
**Intraperitoneal administration of RSV 100 mg/kg daily alone or in combination with cetuximab (10 mg/kg three times weekly)**	Xenografted nude BALB/cAnNCrj-nu/nu mice	Suppression of tumor growth and downregulation of uPAR expression. Synergic effect with cetuximab due to overcoming the drug resistance phenomenon.	Uzawa et al. [44]
**Buccal administration of 74.5 mM of RSV (ethanol solution) or cream and mouthwash containing the RSV–HPCD complex two times a week**	Syrian golden hamsters treated with neoplastic agent	Chemopreventive effects leading to a reduction in OPLs. The RSV–HPCD complex mouthwash highlighted the best efficacy.	Berta et al. [53]
